# A short synthesis of (±)-cherylline dimethyl ether

**DOI:** 10.3762/bjoc.5.80

**Published:** 2009-12-16

**Authors:** Bhima Y Kale, Ananta D Shinde, Swapnil S Sonar, Bapurao B Shingate, Sanjeev Kumar, Samir Ghosh, Soodamani Venugopal, Murlidhar S Shingare

**Affiliations:** 1 Organic Research Laboratory, Department of Chemistry, Dr. Babasaheb Ambedkar Marathwada University, Aurangabad – 431 004 (M.S.), India; 2 Chemistry Section, Applied Sciences and Humanities Department, SVNIT, Surat – 395 007, India

**Keywords:** Curtius rearrangement, Michael reaction, Pictet–Spengler cyclization, radical azidonation

## Abstract

A synthesis of (±)-cherylline dimethyl ether is reported. The key steps involved are Michael-type addition, radical azidonation of an aldehyde, Curtius rearrangement, and reduction of an isocyanate intermediate followed by Pictet–Spengler cyclization.

## Introduction

**Figure 1:**
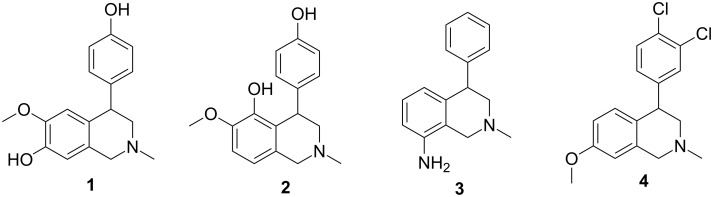
Aryl-1,2,3,4-tetrahydroisoquinolines.

**Scheme 1:**
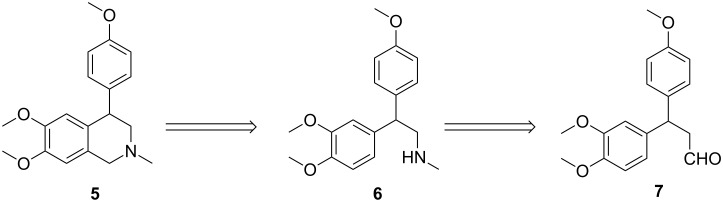
Retrosynthetic analysis of **5**.

Aryl-1,2,3,4-tetrahydroisoquinolines have attracted much attention from the synthetic community owing to the potential biological activities of this class of compounds and their increasing medicinal interest. Among these heterobicyclic compounds cherylline (**1**) (Figure 1), a rare phenolic 4-phenyltetrahydroisoquinoline alkaloid, and its dimethyl ether **5** (Scheme 1). Their structures are unique among the *Amaryllidaceae* alkaloids [1–2] and they have long been fascinating targets for organic chemists as witnessed by a number of articles dealing with biogenesis, isolation, characterization and synthesis. Cherylline (**1**) and latifine (**2**) are the two 4-aryltetrahydroisoquinoline alkaloids isolated from *Amaryllidaceae* plants. Apart from their natural occurence, 4-aryltetrahydroisoquinolines are of interest due to various pharmacological activities [3]. For example, nomifensine (**3**) [3] and dichlofensine (**4**) [4] exhibit CNS activity and inhibit serotonin and dopamine uptake mechanisms.

There are several reports on the syntheses [5–15] of (±)-cherylline and of (±)-latifine [13,16–17] which include some efficient asymmetric syntheses. Most of the reported methods for the synthesis of (±)-cherylline are lengthy. We report herein an alternative efficient synthesis of (±)-cherylline dimethyl ether. The key steps involved are Michael addition, radical azidonation of aldehydes [18], Curtius rearrangement, and reduction of an isocyanate intermediate followed by Pictet–Spengler cyclization.

## Results and Discussion

Our retrosynthetic analysis of (±)-cherylline dimethyl ether (**5**) is depicted in Scheme 1. It can be anticipated that **5** could be constructed via a Pictet–Spengler ring annulation from amine **6** which, in turn, could be obtained by reduction of the corresponding isocyanate. The required isocyanate would arise from the aldehyde **7** via radical azidonation followed by Curtius reaction. Lastly, aldehyde **7** could be assembled by Michael addition of 1,2-dimethoxybenzene to *p*-methoxycinnamonitrile.

**Scheme 2:**
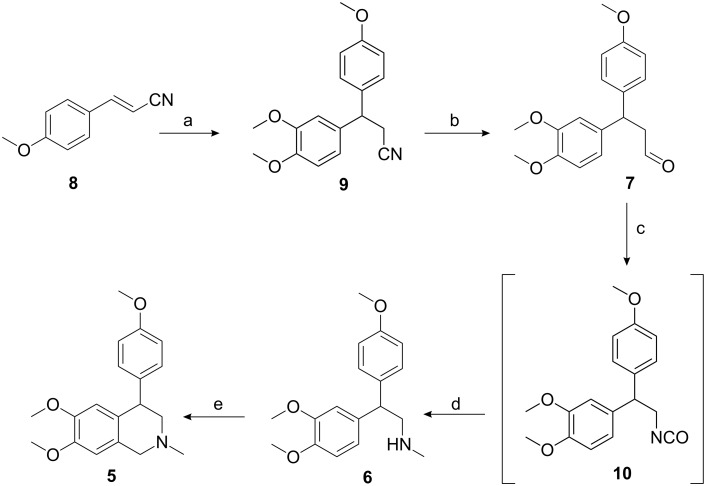
(a) 1,2-Dimethoxybenzene, TFA, reflux, 3 h, 90%; (b) DIBAL-H, CH_2_Cl_2_, −78 °C, 3 h, 65%; (c) (i) NaN_3_, ICl, acetonitrile, CH_2_Cl_2_, 0–5 °C (ii) toluene, 110 °C, 1 h, 65%; (d) LiAlH_4_, THF, reflux, 24 h, 90% (e) HCHO, acetic acid, 90 °C, 2 h, 45%.

1,2-Dimethoxybenzene (veratrole) was subjected to a Michael-type reaction with *p*-methoxycinnamonitrile (**8**) in the presence of trifluoroacetic acid to obtain 3-(3,4-dimethoxyphenyl)-3-(4-methoxyphenyl)propanenitrile (**9**) in 90% yield (Scheme 2). The formation of compound **9** was confirmed by physical spectroscopic data. Reduction of the nitrile group in compound **9** with DIBAL-H in dichloromethane at −78 °C, gave 3-(3,4-dimethoxyphenyl)-3-(4-methoxyphenyl)propanal (**7**) in 70% yield. The conversion of the nitrile to an aldehyde group was confirmed by its IR spectroscopy in which the peak at 1715 cm^−1^ was observed. Radical azidonation of aldehyde **7** with iodine azide generated in situ by the reaction of sodium azide with ICl) at room temperature gave an acyl azide and subsequent Curtius rearrangement provided isocyanate **10** in moderate yield. Immediate reduction of the isocyanate **10** by lithium aluminium hydride in tetrahydrofuran gave *N*-methylamine **6** in 90% yield. The crude amine **6**, on Pictet–Spengler reaction with formaldehyde in acetic acid, gave (±)-cherylline dimethyl ether [15] **5** in 45% yield.

In short, we have devised a short and efficient method for the synthesis of (±)-cherylline dimethyl ether. The simple and facile nature of this tetrahydroisoquinoline synthesis should allow the construction of a wide variety of interesting and useful analogous molecules.

## Experimental

### Materials and Instruments

All solvents and reagents were purchased from the suppliers and used without further purification. All non-aqueous reactions were performed in dry glassware under an atmosphere of dry nitrogen. Organic solutions were concentrated under reduced pressure. Thin layer chromatography was performed on Merck precoated Silica-gel 60F_254_ plates. ^1^H and ^13^C NMR spectra were recorded in DMSO-*d*_6_ and CDCl_3_ using 400 MHz, on a Varian Gemini 400 MHz FT NMR spectrometer. The chemical shifts were reported in δ ppm relative to TMS. The IR spectra were recorded in the solid state as KBr dispersion using Perkin Elmer FT-IR spectrophotometer. The mass spectra were recorded on Shimadzu LCMS-QP 800 LC-MS and AB-4000 Q-trap LC-MS/MS. Melting points were obtained by using the open capillary method and are uncorrected.

**3-(3,4-Dimethoxyphenyl)-3-(4-methoxyphenyl)propanenitrile (9).** A solution of *p*-methoxycinnamonitrile (6.0 g, 0.037 mol) and veratrole (10.4 g, 0.75 mol) in trifluoroacetic acid (TFA) (30 mL) was refluxed for 3 h. TFA was removed by evaporation under reduced pressure and saturated NaHCO_3_ (50 mL) was added. The mixture was extracted with ethyl acetate (3 × 30 mL) and combined extracts were dried over sodium sulfate and concentrated. The residue was purified by column chromatography on silica gel (7:3 hexane and ethyl acetate) to yield **9** as a residue (10 g, 90 %); IR (KBr, cm^−1^): 2240 (CN); ^1^H NMR (CDCl_3_) (δ ppm): 2.96 (2H, d, *J* = 8.0 Hz, –CH–C*H_2_*–), 3.76 (3H, s, –OCH_3_), 3.79 (3H, s, –OCH_3_), 3.83 (3H, s, –OCH_3_), 4.25 (1H, t, *J* = 8.0 Hz, C*H*–CH_2_), 6.65–7.12 (7H, m, ArH); ^13^C NMR (CDCl_3_) (δ ppm); 23.6, 44.5, 55.1, 55.8, 56.1, 110.9, 114.9, 119.4, 121.4, 128.4, 132.9, 133.0, 148.3, 148.8, 157.6; MS (m/z): 298 [M^+^ + 1].

**3-(3,4-Dimethoxyphenyl)-3-(4-methoxyphenyl)propanal (7).** To a solution of 3-(3,4-dimethoxyphenyl)-3-(4-methoxyphenyl)propanenitrile (4.0 g, 0.012 mol) in dichloromethane (40 mL) at −78 °C was added dropwise DIBAL-H (1.0 M in CH_2_Cl_2_, 36.0 mL, 0.036 mol). The reaction mixture stirred at −78 °C for 2–3 h. Saturated aqueous solution of Rochelle’s salt was added and it was stirred vigorously at room temperature until the two layers became clear. The mixture was extracted with ethyl acetate (3 × 50 mL) and combined extracts were dried over anhydrous sodium sulfate and concentrated. The residue was purified by column chromatography on silica gel (7:3 hexane and ethyl acetate) to give aldehyde **7** as yellow oil (2.5 g, 65%); IR (KBr, cm^−1^): 1725; ^1^H NMR (CDCl_3_) (δ ppm): 3.09 (2H, d, *J* = 7.8 Hz, –CH–C*H_2_*–), 3.77 (3H, s, –OCH_3_), 3.82 (3H, s, –OCH_3_), 3.84 (3H, s, –OCH_3_), 4.52 (1H, t, *J* = 7.8 Hz, C*H*–CH_2_), 6.69–7.14 (7H, m, ArH), 9.72 (1H, t, –CHO); ^13^C NMR (CDCl_3_) (δ ppm); δ 43.5 (CH_2_), 49.0 (Ar–CH–Ar), 55.4 (OCH_3_), 55.8 (OCH_3_), 55.8 (OCH_3_), 111.9 (CH_ar_), 112.1 (CH_ar_), 114.1 (2 × CH_ar_), 119.6 (CH_ar_) 128.8 (2 × CH_ar_), 136.6 (C_ar_), 137.2 (C_ar_), 147.6 (OC_ar_), 149.0 (OC_ar_), 158.0 (OC_ar_), 202.8 (CHO). MS (m/z): 299 [M^+^− H], 323 [M^+^ + Na].

**2-(3,4-Dimethoxyphenyl)-2-(4-methoxyphenyl)-*N*-methylethanamine (6).** To a stirred slurry of sodium azide (1.3 g, 0.02 mol) in acetonitrile (30 mL) in an ice bath was added slowly iodine monochloride (2.16 g, 0.01 mol) in acetonitrile (30 mL). The reaction mixture was stirred for an additional 5–10 min and 3-(3,4-dimethoxyphenyl)-3-(4-methoxyphenyl)propanal (2.0 g, 0.006 mol) in acetonitrile (10 mL) was added. The reaction was allowed to reach room temperature and was stirred for 2.5 h. The red–brown slurry was poured over water (50 mL), and the mixture was extracted with dichloromethane (100 mL). The organic extracts were combined and washed with 3 × 50 mL of 5% sodium thiosulfate leaving a colorless solution, which was dried over anhydrous magnesium sulfate. Removal of the solvent under reduced pressure produced acyl azide.

**Curtius rearrangement**. The acyl azide was dissolved in dry toluene (30 mL) and heated to reflux for 1 h. Concentration of reaction mixture under vacuum gave isocyanate **10** (1.25 g, 65%) . IR (KBr, cm^−1^): 2260 (N=C=O). Isocyanate **10** (1.25 g, 0.003 mol) in dry THF (15 mL) was slowly added to the suspension of lithium aluminium hydride (LAH) (0.6 g, 0.015 mol) in THF (15 mL) under nitrogen atmosphere. After refluxing the reaction mixture for 24 h, it was cooled to 5 °C and cold water was slowly added to it. The aluminium hydroxide formed was filtered over celite and washed with chloroform. The filtrate was extracted with chloroform (3 × 20 mL). All the organic extracts and washings were combined, dried over anhydrous sodium sulfate, concentrated to obtain **6** as a brown residue 1.0 g (90%); IR (film, cm^−1^): 3120 (NH); ^1^H NMR (CDCl_3_) (δ ppm): 2.44 (3H, s, NCH_3_), 3.12 (2H, d, *J* = 8.0 Hz, HCH–N–CH_3_), 3.78 (3H, s, OCH_3_), 3.83 (3H, s, OCH_3_), 3.84 (3H, s, OCH_3_), 4.08 (1H, t, *J* = 7.6 Hz, Ar–CH–Ar), 6.74–7.26 (7H, m, ArH); ^13^C NMR (CDCl_3_) (δ ppm); 43.9 (NCH_3_), 49.4 (ArCHAr), 55.3 (OCH_3_), 55.8 (OCH_3_), 55.9 (OCH_3_), 56.6 (NCH_2_Ar), 112.1 (CH_ar_), 112.2 (CH_ar_), 114.1 (2 × CH_ar_), 119.8 (C_ar_), 129.0 (2 × C_ar_), 136.2 (C_ar_), 136.8 (C_ar_), 147.5 (OC_ar_), 148.9 (OC_ar_),157.9 (OC_ar_). HRMS m/z calculated for C_18_H_23_NO_3_ 302.3801 [M+1], found: 302.37.

**(±)-Cherylline dimethyl ether (5).** A mixture of **6** (2.0 g, 0.006 mol), formaldehyde (0.64 g, 0.007 mol) and acetic acid (5 mL) was stirred at 90 °C under nitrogen atmosphere for 2 h. After cooling to room temperature, the reaction mixture was basified using saturated NaHCO_3_ solution. This basified solution was extracted ethyl acetate (3 × 25 mL), dried, and concentrated to obtain 1.1 g of crude product. Purification of crude product by column chromatography using 1% methanol in dichloromethane as an eluent afforded compound **5** (0.93 g, 45%); IR (KBr, cm^−1^): 1610, 1514. ^1^H NMR (CDCl_3_) (δ ppm) : 2.41 (3H, s, NCH_3_), 2.48 (1H, s (br), Ar–*H*CH–N), 2.98 (1H, s (br), Ar–HC*H*–N), 3.56 (2H, s (br), CH–C*H_2_*–N), 3.68 (3H, s, OCH_3_), 3.76 (3H, s, OCH_3_), 3.82 (3H, s, OCH_3_), 4.12 (1H, s (br), Ar–C*H*–Ar), 6.31 (1H, s, 5–CH), 6.52 (1H, s, 8–CH), 6.82 (2H, d, *J* = 8.4 Hz, 2′–CH), 7.08 (2H, d, *J* = 8.4 Hz, 3′–CH). ^13^C NMR (CDCl_3_) (δ ppm); 43.9 (NCH_3_), 45.9 (ArCHAr), 55.3 (OCH_3_), 55.8 (OCH_3_), 55.9 (OCH_3_), 57.7 (NCH_2_–), 61.6 (NCH_2_Ar), 109.7 (CH_ar_), 112.5 (CH_ar_), 113.9 (2 × CH_ar_), 127.7 (C_ar_), 129.2 (C_ar_), 130.0 (2 × C_ar_), 137.7 (C_ar_), 147.5 (OC_ar_), 147.6 (OC_ar_), 158.0 (OC_ar_). HRMS m/z calculated for C_19_H_23_NO_3_ 314.1756 [M+1], found: 314.175.
